# Self-reported risky sexual practices among adolescents and young adults in Botswana

**DOI:** 10.4102/sajhivmed.v20i1.899

**Published:** 2019-06-26

**Authors:** Unoda Chakalisa, Kathleen Wirth, Kara Bennett, Etienne Kadima, Kutlo Manyake, Tendani Gaolathe, Pam Bachanas, Tafireyi Marukutira, Refeletswe Lebelonyane, Scott Dryden-Peterson, Lisa Butler, Mompati Mmalane, Joseph Makhema, Michelle E. Roland, Molly Pretorius-Holme, Max Essex, Shahin Lockman, Kathleen M. Powis

**Affiliations:** 1 Botswana Harvard AIDS Institute Partnership, Gaborone, Botswana; 2 Harvard T.H. Chan School of Public Health, Boston, United States; 3 Bennett Statistical Consulting, Ballston Lake, United States; 4 Centers for Disease Control and Prevention, Division of Global HIV/AIDS and TB, Atlanta, United States; 5 Centers for Disease Control and Prevention, Gaborone, Botswana; 6 Botswana Ministry of Health and Wellness, Gaborone, Botswana; 7 Brigham and Women’s Hospital, Boston, United States; 8 Institute for Collaboration on Health, Intervention and Policy, University of Connecticut, Storrs, United States; 9 Massachusetts General Hospital, Boston, United States

**Keywords:** Adolescents, Young adults, Risky sexual practices, HIV, Self-reported risky sexual practices, Gender-specific risky sexual practices

## Abstract

**Background:**

Adolescents and young adults account for more than one-third of incident Human Immunodeficiency Virus (HIV) infections globally. Understanding sexual practices of this high-risk group is critical in designing HIV targeted prevention programming.

**Objectives:**

To describe self-reported risky sexual practices of adolescents and young adults aged 16–24 years from 30 Botswana communities.

**Methods:**

Cross-sectional, self-reported age at sexual debut; number of sexual partners; condom and alcohol use during sex; intergenerational sex; and transactional sex data were collected. Modified Poisson estimating equations were used to obtain univariate and multivariate-adjusted prevalence ratios (PR) and 95% confidence intervals (CI) comparing engagement in different sexual practices according to gender, accounting for the clustered design of the study.

**Results:**

Among the 3380 participants, 2311 reported being sexually active with more females reporting being sexually active compared to males (65% vs. 35%, respectively; *p* < 0.0001). In univariate analyses, female participants were more likely to report inconsistent condom use (PR 1.61; 95% CI 1.44–1.80), intergenerational sex (PR 9.00; 95% CI 5.84–13.88) and transactional sex (PR 3.46; 95% CI 2.07–5.77) than males, yet less likely to report engaging in sex before age 15 years (PR 0.59; 95% CI: 0.41–0.85), using alcohol around the time of intercourse (PR: 0.59; 95% CI 0.45–0.76) or having ≥ two partners in the last 12 months (PR 0.65; 95% CI 0.57–0.74).

**Conclusions:**

Self-reported risky sexual practices of adolescents and young adults in Botswana differed significantly between males and females. Gender-specific risky sexual practices highlight the importance of developing tailored HIV prevention programming.

## Introduction

According to the United Nations International Children’s Emergency Fund (UNICEF), every 2 minutes an adolescent aged 15–19 years is infected with Human Immunodeficiency Virus (HIV), with female adolescents and young adults aged 15–24 years in sub-Saharan Africa at the greatest risk.^1,2^ The World Health Organization (WHO) estimates that 20% of women living with HIV worldwide are aged 15–24 years, of whom 80% live in sub-Saharan Africa.^2,3^ AIDS remains one of the leading causes of death among adolescents worldwide, with sub-Saharan Africa the most affected.^1,4^

Human immunodeficiency virus prevention programming represents a critical component of HIV epidemic eradication. However, prevention programme designs and offerings need to reflect the dynamics of the epidemic. For example, females acquire HIV approximately 5–7 years earlier than their male counterparts in sub-Saharan Africa.^5^ Factors contributing to this gender disparity include behavioural differences, such as challenges around the ability to negotiate condom use, or vulnerability to incidents of forced sex, as well as structural differences, including poverty, differential livelihood opportunities, and unequal access to higher education.^6,7^ Therefore, contextually and age-appropriate prevention programming that effectively reaches adolescents and young adults in sub-Saharan Africa (especially women) is urgently needed.

Using baseline (pre-intervention) data collected in the YaTsie study, a large community-based HIV prevention study, we analysed self-reported sexual practices in a large population-based random sample of adolescents and young adults aged 16–24 years from 30 communities in rural and peri-urban Botswana to describe sexual practices that place individuals at risk of HIV acquisition. We evaluated self-reported risky sexual practices by gender and modelled predictors of these practices.

## Methods

### Study design and population

YaTsie, also known as the Botswana Combination Prevention Project (clinicaltrials.gov NCT01965470), is a pair-matched, cluster-randomised study, funded by the United States President’s Emergency Plan for AIDS Relief (PEPFAR).^8^ The study was designed to determine whether implementation of a package of combination prevention interventions reduces population-level cumulative 3-year HIV incidence.

The trial, which began in October 2013, was conducted in 30 rural and peri-urban communities in Botswana (15 matched pairs), with a combined population of approximately 180 000 people, representing nearly 10% of Botswana’s total estimated population. Fifteen communities were randomised to a combination prevention intervention package with HIV testing, linkage to care, expanded ART and male circumcision components. Fifteen communities were randomised to a non-intervention group where standard of care was maintained.^8^ Prior to initiation of the interventions, we enrolled a prospective cohort to measure HIV incidence and intervention uptake over time (main objectives of the parent study). The survey participants were community residents aged 16–64 years, recruited from a random sample of approximately 20% of households in each study community. Refer to prior publications for more details on sampling approach in the parent study.^8^ A total of 12 610 consenting or assenting household residents were enrolled into the main study between October, 2013, and November, 2015. For inclusion in the analysis of risky sexual practices, individuals aged 16–24 years must have provided consent or assent for the YaTsie parent study and completed the risky sexual practices questionnaire, a questionnaire that could be completed at the discretion of the study participant.

### Data collection

This analysis uses only initial cross-sectional data from responding participants in the first household survey conducted prior to initiation of interventions. Research assistants administered structured case report forms to collect data on sexual practices. All participants were asked whether or not they had ever engaged in sexual intercourse. Among those who reported being sexually active, further questions were asked of the participant, including age of sexual debut, specifically about sexual activity in the last 12 months, number of sexual partners, intergenerational sex, transactional sex, alcohol use during intercourse either by the respondent or the respondent’s partner and consistency of condom use either by self or partner. All respondents were asked about their HIV testing history and asked to provide documentation of prior tests (medical records with written test results or antiretroviral treatment prescriptions). Any individual who was not known to be HIV-positive (with supporting documentation) was asked to undergo HIV testing and counselling during home visits. Demographic information was collected from each participant, including information on education level, employment, income, water source in home, access to electricity and household commodities.

### Statistical analysis

We *a priori* defined risky sexual practices as self-reported participation in any of the following: sexual debut before 15 years of age, sexual intercourse with a partner older than the respondent by 10 years or more (intergenerational sex); receipt of money, transport, food, drink or other goods in exchange for sex (transactional sex); any alcohol use (by the respondent, the respondent’s partner or both) during sexual intercourse; and more than one occasion of not using a condom by the respondent or the respondent’s partner (inconsistent condom use) over the preceding 12 months.

We compared the socio-demographic characteristics of adolescents and young adults who reported being sexually active and those who were not, using a Wald χ^2^ test. Modified Poisson estimating equations were used to obtain prevalence ratios (PR) for engagement in risky sexual practices according to gender, adjusting for community-level clustering. Modified Poisson regression, as opposed to logistic regression, was necessary given the overall commonality of the outcomes of interest. Specifically, we note that in settings such as ours where the outcome is not rare (i.e. > 10%), the odds ratio estimated by logistic regression will be an upwardly biased estimate of the underlying risk ratio. For risky sexual practices found to differ significantly between male and female respondents, gender-specific multivariate models were constructed by including all covariates with a *p*-value ≤ 0.10 in univariate analyses. All analyses were conducted using SAS, version 9.3 (SAS Institute, Cary, North Carolina, USA), assuming a two-sided, 95% confidence interval (CI).

We used descriptive statistics to report the proportion of adolescents and young adults with presumed perinatal acquisition of HIV, defining perinatal HIV acquisition as documentation of HIV diagnosis before 10 years of age. Using Fisher’s exact tests, we compared HIV testing history among sexually active adolescents and young adults by gender, known HIV diagnosis prior to the BCPP baseline survey and by self-reported risky sexual practices, comparing those reporting ≥ two risky sexual practices to those with ≤ one.

## Ethical consideration

The study protocol, informed consent and other materials were approved by the Botswana Health Research Development Committee, and the US Centers for Disease Control and Prevention, the two governing Institutional Review Boards for the BCPP study. Written informed consent was obtained from all participants aged 18 years and above, while participants aged 16–17 years provided written assent, with parents or guardians providing written permission.

## Results

Among the 12 610 individuals who consented or assented with parental permission to participate in the parent study at the initial baseline visit, 3413 were adolescents and young adults aged 16–24 years. A total of 3380 responded to the sexual activity questionnaire, with 2311 (68%) reporting prior engagement in sexual activity and 1069 (31%) reporting never having been sexually active. The majority (~80%) of participants reported a secondary level education. A higher proportion of sexually active individuals reported lack of television (44% vs. 37%; *p* < 0.0001) or refrigeration (55% vs. 46%; *p* < 0.0001) and reliance on a communal stand pipe for water (26% vs. 20%; *p* < 0.0001).

Female participants were significantly more likely to report ever being sexually active than males (76% vs 58%, respectively; *p* < 0.0001) (Table 1). Among all adolescents and young adults, regardless of history of being sexually active, 93% were HIV-uninfected at the time of the survey. Among the 1069 individuals reporting no prior sexual activity, 36 (3%) were HIV-infected, of whom 30 (83%) were adolescents, whereas 184 (8%) individuals reporting prior sexual activity were HIV-infected, with 85% of the infections occurring in young adults aged 20–24 years. Twelve (33%) of the individuals with HIV who reported no prior sexual activity had documentation of diagnosis prior to 10 years of age, suggesting perinatal acquisition of HIV, while nine (25%) were newly diagnosed with HIV during the YaTsie baseline household HIV testing.

**TABLE 1:** Summary of socio-demographic characteristics of adolescents and young adults (aged 16–24 years) by gender.

Characteristic (*n*)†	Males (*n* = 1417)		Females (*n* = 1963)		*p*
	*n*	%	*n*	%	
**Education level (*n* = 3373)**					**0.02**
None or primary	89	6	92	5	
Secondary	1147	81	1562	80	
Tertiary	180	13	303	15	
**Sexually active (*n* = 3380)**	815	58	1496	76	**< 0.0001**
**HIV status (*n* = 3372)**					**< 0.0001**
Positive	37	3	183	9	
Negative	1377	97	1775	91	
**Household food insecurity (*n* = 3334)**					0.62
Never	960	69	1358	70	
Rarely	198	14	247	13	
Sometimes	182	13	250	13	
Often	55	4	84	4	
**Refrigeration in household (*n* = 3165)**	641	51	863	47	0.28
**Cooking source in household (*n* = 3336)**					0.10
Gas	356	26	444	23	
Electricity	235	17	336	17	
Charcoal/wood	799	57	1144	59	
Other	5	< 1	17	1	
**Water source (*n* = 3335)**					0.11
Piped indoors	169	12	255	13	
Standpipe or tap in yard	888	64	1155	60	
Communal stand pipe	306	22	483	25	
Other	32	2	47	2	
**Toilet type (*n* = 3336)**					0.09
Flush toilet in home	230	17	340	18	
Pit latrine in yard	1021	73	1366	70	
Communal toilet/pit latrine	100	7	143	7	
Bush or other	44	3	92	5	
**Television in home (*n* = 3165)**	788	40	1044	57	0.07
**Access to internet (*n* = 3165)**	169	13	237	13	0.99
**Own a cell phone (*n* = 3165)**	1252	95	1747	95	0.51
**Community population size in quartiles (*n* = 3380)**					0.68
2700 to 3899 persons	223	16	332	17	
3900 to 5199 persons	360	25	502	26	
5200 to 7499 persons	378	27	493	25	
7500 to 12 850 persons	456	32	636	32	
**Distance from an urban area, in quartiles (*n* = 3380)**					0.99
18 to 24 km	268	19	377	19	
24 to 40 km	467	33	640	33	
41 to 84 km	246	17	340	17	
85 to 380 km	436	31	606	31	

† , ‘n’ of < 3380 for any socio-demographic variable reflects lack of response from the participant to that question.

### Risky sexual practices by gender

Among the sexually active respondents, we evaluated risky sexual practices by gender (Table 2). Compared with males, females were significantly more likely to report inconsistent condom use (PR 1.61; 95% CI 1.44–1.80), intergenerational sex (PR 9.00; 95% CI 5.84–13.88) and transactional sex (PR3.46; 95% CI 2.07–5.77) during the prior 12 months. However, female participants were significantly less likely to report having engaged in sex before 15 years of age (PR 0.59; 95% CI 0.41–0.85), report use of alcohol (by either partner) during sexual intercourse (PR 0.59; 95% CI 0.45–0.76) or having ≥ two partners in the last 12 months (PR 0.65; 95% CI 0.57–0.74).

**TABLE 2:** Summary of risky sexual practices among sexually active adolescents and young adults by gender.

Risky sexual practice (*n* with data)	*N* (%)				for female gender (vs. males)		*P*
	Males (*N* = 815)		Females (*N* = 1496)				
	*n*	%	*n*	%	PR	95% CI	
Sexual debut before age 15 years (*n* = 2311)	119	15	129	9	0.59	0.41–0.85	0.01
Alcohol use by respondent, partner or both during intercourse (*n* = 2308)	93	11	100	7	0.59	0.45–0.76	< 0.0001
Inconsistent condom use (*n* = 2012)	237	29	742	50	1.61	1.44–1.80	< 0.0001
≥Two sex partners in the last 12 months (*n* = 2247)	332	42	395	27	0.65	0.57–0.74	< 0.0001
Intergenerational sex with older partner (*n* = 2028)	21	3	373	25	9.00	5.84–13.88	< 0.0001
Transactional sex (*n* = 2048)	18	2	122	8	3.46	2.07–5.77	< 0.0001

Note: A PR of > 1 indicates that the ratio of females reporting a specific sexual practice exceeds that of males. For example, self-reported inconsistent condom use was 61% more prevalent among females than males, whereas a PR of < 1 indicates that fewer females reported the practices compared to males.

CI, confidence interval; PR, prevalence ratio.

### Predictors of gender-specific engagement in high-risk sexual practices

We modelled factors associated with risky sexual practices, stratified by gender. For females, factors associated with consistent condom use, intergenerational sex with a partner ≥ 10 years older and transactional sex were modelled. For males, factors associated with early sexual debut, alcohol use with sex and ≥ two sex partners in the last 12 months were modelled (Table 3). Young adult females aged 20–24 years were 30% (PR 1.30; 95% CI 1.13–1.49) more likely to report inconsistent condom use compared with adolescent females aged 16–19 years. In multivariate analysis, a positive HIV status was associated with intergenerational sex among female participants (PR 1.29; 95% CI 1.00–1.67). Reporting occasional food insecurity (PR 2.04; 95% CI 1.35–3.09) and lack of a cell phone (PR 2.12; 95% CI 1.32–3.40) were associated with higher prevalence of engagement in transactional sex among females.

**TABLE 3:** Univariate and multivariate modelling factors for risky sexual practices among female adolescents and young adults.

Risk factor	Inconsistent condom use				Intergenerational sex with older partner				Transactional sex			
	Univariate model		Multivariate model^†^		Univariate model		Multivariate model^‡^		Univariate model		Multivariate model^§^	
	Prevalence ratio	*p*	Prevalence ratio	*p*	Prevalence ratio	*p*	Prevalence ratio	*p*	Prevalence ratio	*p*	Prevalence ratio	*p*
**Age**	-	**< 0.001**	-	**< 0.01**	-	0.57	-	-	-	0.51	-	-
Adolescent (16–19)	REF	-	REF	-	REF	-	-	-	REF	-	-	-
Young adult (20–24)	1.28 (1.12–1.47)	-	1.30 (1.13–1.49)	-	1.08 (0.83–1.40)	-	-	-	1.14 (0.77–1.69)	-	-	-
**HIV status¶**	-	0.10	-	0.09	-	0.06	-	**0.05**	-	0.41	-	-
Negative	REF	-	REF	-	REF	-	REF	-	REF	-	-	-
Positive	0.88 (0.75–1.03)	-	0.87 (0.74–1.02)	-	1.29 (1.00–1.67)	-	1.31(1.00–1.72)	-	1.22 (0.76–1.96)	-	-	-
**Smaller meals**	-	0.10	-	0.16	-	0.59	-	-	-	**0.02**	-	**0.02**
Never	REF	-	REF	-	REF	-	-	-	REF	-	REF	-
Rarely	1.01 (0.90–1.13)	-	0.99 (0.89–1.11)	-	0.92 (0.69–1.22)	-	-	-	2.02 (1.31–3.10)	-	2.04 (1.35–3.09)	-
Sometimes/often	1.17 (1.04–1.32)	-	1.15 (1.02–1.31)	-	1.08 (0.90–1.29)	-	-	-	0.8 (0.44–1.55)	-	0.89 (0.47–1.65)	-
**Refrigerator in home**	-	**0.04**	-	0.11	-	0.19	-	-	-	0.98	-	-
Yes	REF	-	REF	-	REF	-	-	-	REF	-	-	-
No	1.11 (1.00–1.23)	-	1.09 (0.98–1.21)	-	0.90 (0.76–1.06)	-	-	-	0.99 (0.66–1.50)	-	-	-
**Water source**	-	0.21	-	-	-	0.07	-	0.06	-	0.88	-	-
Piped into home	REF	-	-	-	REF	-	REF	-	REF	-	-	-
Tap/stand pipe on plot	0.92 (0.75–1.12)	-	-	-	1.01 (0.77–1.34)	-	1.01 (0.76–1.34)	-	1.15 (0.63–2.10)	-	-	-
Communal/other	1.01 (0.85–1.20)	-	-	-	0.84 (0.67–1.05)	-	0.81 (0.64–1.03)	-	1.06 (0.54–2.08)	-	-	-
**Cell phone**	-	0.36	-	-	-	0.06	-	0.12	-	**<0.001**	-	**<0.001**
Yes	REF	-	-	-	REF	-	REF	-	REF	-	REF	-
No	0.89 (0.7–1.15)	-	-	-	1.35 (0.98–1.84)	-	1.29 (0.93–1.78)	-	2.14 (1.40–3.27)	-	2.12 (1.32–3.40)	-
**Internet access at home**		0.28	-	-	-	0.46	-	-	-	0.44	-	-
Yes	REF	-	-	-	REF		-	-	REF	-	-	-
No	0.95 (0.85–1.05)	-	-	-	1.13 (0.82–1.56)	-	-	-	1.37 (0.61–3.08)	-	-	-
**Community pop quartile**		0.81	-	-	-	0.48	-	-	-	0.97	-	-
1st (2700–3899)	REF	-	-	-	REF	-	-	-	REF	-	-	-
2nd (3900–5199)	0.94 (0.82–1.09)	-	-	-	1.00 (0.80–1.26)	-	-	-	1.13 (0.66–1.94)	-	-	-
3rd (5200–7499)	0.98 (0.83–1.16)	-	-	-	1.20 (0.99–1.47)	-	-	-	1.12 (0.56–2.22)	-	-	-
4th (7500–12 850)	0.95 (0.82–1.09)	-	-	-	1.05 (0.90–1.22)	-	-	-	1.11 (0.67–1.85)	-	-	-
**Distance from urban area**		0.36	-	-		0.64	-	-	-	0.24	-	-
1st (18–24)	0.85 (0.69–1.05)	-	-	-	0.95 (0.80–1.12)	-	-	-	0.70 (0.36–1.36)	-	-	-
2nd (24–40)	0.92 (0.81–1.05)	-	-	-	0.92 (0.74–1.14)	-	-	-	1.19 (0.81–1.74)	-	-	-
3rd (41–84)	0.88 (0.71–1.09)	-	-	-	0.86 (0.69–1.07)	-	-	-	0.62 (0.32–1.23)	-	-	-
4th (85–380)	REF	-	-	-	REF	-	-	-	REF	-	-	-

† , Multivariate model of inconsistent condom use adjusted for HIV status, food insecurity and lack of refrigerator in the home.

‡ , Multivariate model of transactional sex adjusted for HIV status, household water source and respondent’s ownership of a cell phone.

§ , Multivariate model of intergenerational sex food insecurity and respondent’s ownership of a cell phone.

¶ , HIV status at time of baseline household survey with in home testing (May not have known their positive status prior to answering questions on sexual practices).

Factors associated with risky sexual practices among males are presented in Table 4. In univariate analysis, HIV infection was associated with sexual debut before 15 years of age, with males living in proximity to an urban setting more likely to be HIV-infected as opposed to those living in proximity to a rural setting. These two significant relationships held up in multivariate analysis. The adjusted PR in multivariate analysis for HIV-infected adolescents and young adults compared with HIV-uninfected adolescents and young adults was over three-fold higher (PR 3.28; 95% CI 2.05–5.21), while living closer to an urban setting was protective against early sexual debut (PR 0.39; 95% 0.24–0.66). Young adult males aged to 20–24 years were significantly more likely to report alcohol use with sex compared with adolescent males aged 16–19 years (PR 2.07; 95% CI 1.12–3.82). In evaluating factors associated with having ≥ two partners in the last 12 months, after controlling for community population quartile, males with internet access experienced a significantly higher PR of reporting ≥ two partners in the last year, with lack of access to internet being protective (PR:0.74; 95% CI 0.62–0.89).

**TABLE 4:** Univariate and multivariate modelling of factors for risky sexual practices among male adolescents and young adults.

Risk factor	Early sexual debut				Alcohol use with sex				≥ Two sex partners in the last 12 months			
	Univariate model		Multivariate model†		Univariate model		Multivariate model		Univariate model		Multivariate model‡	
	Prevalence ratio	*p*	Prevalence ratio	*p*	Prevalence ratio	*p*	Prevalence ratio	*p*	Prevalence ratio	*p*	Prevalence ratio	*p*
**Age**	-	0.36	-	-	-	**0.02**	-	**0.02**	-	0.12	-	-
Adolescent (16–19)	REF	-	-	-	REF	-	REF	-	REF	-	-	-
Young adult (20–24)	1.23 (0.79–1.92)	-	-	-	2.07 (1.12–3.82)	-	2.07 (1.12–3.82)	-	1.15 (0.97–1.37)	-	-	-
**HIV status**§	-	**<0.01**	-	<**0.000**	-	0.45	-	-		0.84	-	-
Negative	REF	-	REF	**1**	REF	-	-	-	REF	-	-	-
Positive	2.78 (1.69–4.58)	-	3.28 (2.05–5.21)	**-**	0.48 (0.07–3.28)	-	-	-	0.93 (0.45–1.92)	-	-	-
**Smaller meals**	-	0.20	-	**-**	-	0.56	-	-		0.92	-	-
Never	REF	-	-		REF	-	-	-	REF	-	-	-
Rarely	0.52 (0.25–1.12)	-	-	-	0.92 (0.48–1.77)	-	-	-	1.04 (0.87–1.24)	-	-	-
Sometimes/often	0.99 (0.70–1.40)	-	-	-	1.29 (0.84–1.96)	-	-	-	1.00 (0.84–1.19)	-	-	-
**Refrigerator in home**	-	0.57	-	-	-	0.36	-	-		0.46	-	-
Yes	REF	-	-	-	REF	-	-	-	REF	-	-	-
No	1.09 (0.81–1.47)	-	-	-	1.28 (0.76–2.15)	-	-	-	1.07 (0.90–1.27)	-	-	-
**Water source**	-	0.39	-	-	-	0.37	-	-		0.42	-	-
Piped into home	REF	-	-	-	REF	-	-	-	REF	-	-	-
Tap/standpipe on plot	0.98 (0.66–1.45)	-	-	-	1.38 (0.77–2.48)	-	-	-	1.02 (0.80–1.31)	-	-	-
Communal/other	1.29 (0.84–1.96)	-	-	-	1.73 (0.85–3.55)	-	-	-	0.87 (0.65–1.16)	-	-	-
**Cell phone**	-	0.10	-	0.08	-	0.75	-	-		0.52	-	-
Yes	REF	-	REF	-	REF	-	-	-	REF	-	-	-
No	0.35 (0.10–1.23)	-	0.36 (0.12–1.12)	-	0.85 (0.30–2.40)	-	-	-	0.88 (0.60–1.30)	-	-	-
**Internet access at home**	-	0.12	-	-	-	0.81	-	-	-	**< 0.0001**	-	**< 0.0001**
Yes	REF	-	-	-	REF	-	-	-	REF	-	REF	-
No	0.69 (0.43–1.10)	-	-	-	1.05 (0.70–1.58)	-	-	-	0.74 (0.62–0.89)	-	0.74 (0.62–0.88)	-
**Community pop. quartile**	-	0.29	-	-	-	0.24	-	-	-	0.10	-	0.09
1st (2700–3899)	REF	-	-	-	REF	-	-	-	REF	-	REF	-
2nd (3900–5199)	0.72 (0.37–1.41)	--	-	-	2.29 (1.02–5.17)	-	-	-	0.79 (0.63–0.99)	-	0.79 (0.61–1.03)	-
3rd (5200–7499)	0.46 (0.23–0.91)	-	-	-	1.44 (0.58–3.57)	-	-	-	0.88 (0.77–0.99)	-	0.84 (0.71–0.98)	-
4th (7500–12 850)	0.46 (0.23–0.95)	-	-	-	1.05 (1.03–5.01)	-	-	-	1.03 (0.86–1.24)	-	1.02 (0.83–1.25)	-
**Distance from urban area**	-	**0.04**	-	**0.02**	0.28	-	-		0.20	-	-
1st (18–24)	0.37 (0.24–0.58)	-	0.39 (0.24–0.66)	-	0.90 (0.48–1.67)	-	-	-	1.13 (0.87–1.46)	-	-	-
2nd (24–40)	0.47 (0.28–0.79)	-	0.55 (0.36–0.85)	-	1.66 (0.82–3.38)	-	-	-	1.22 (1.01–1.47)	-	-	-
3rd (41–84)	0.32 (0.15–0.66)	-	0.33 (0.14–0.80)	-	1.06 (0.47–2.38)	-	-	-	1.01 (0.80–1.28)	-	-	-
4th (85–380)	REF	-	REF	-	REF	-	-	-	REF	-	-	-

† , Multivariate model of early sexual debut adjusted for HIV status, respondent’s ownership of a cell phone and distance from an urban setting.

‡ , Multivariate model of having ≥two sexual partners in the last 12 months adjusted for respondent’s access to internet at home and community size in population quartiles.

§ , HIV status at time of baseline household survey with in home testing (may not have known their positive status prior to answering questions on sexual practices).

### Human immunodeficiency virus testing practices and Human Immunodeficiency Virus prevalence among sexually active adolescents and young adults

In total, 99% of adolescents and young adults who did not have documentation of being HIV-infected accepted in home HIV testing and counselling during the initial household survey. Adolescent and young adult females were significantly more likely to have been diagnosed with HIV than males prior to the initial YaTsie survey (20% vs. 4%, respectively; *p* < 0.0001). In addition, a significantly higher proportion of females than males were newly diagnosed with HIV (5% vs. 2%, respectively; *p* < 0.0001). Among the 2203 adolescents and young adults (1394 females and 809 males) who reported being sexually active but who were not known to be HIV-infected prior to the initial YaTsie household survey, adolescent and young adult females were significantly more likely to have documentation of undergoing HIV testing in the 12 months prior to the survey compared with males (50% vs. 29%, respectively; *p* < 0.0001).

Among adolescent females, 44% (148) reported having at least one child or currently being pregnant. This rate was higher among young adult females at 73% (842). Among adolescent females, 89% of those with a child or who were currently pregnant reported having previously tested for HIV, compared with only 47% of nulliparous female adolescents (*p* < 0.0001). Among young adult females, 95% of those with a child or who were currently pregnant reported having previously participated in HIV testing, compared with 71% of nulliparous female young adults (*p* < 0.0001).

There was a trend towards increased HIV prevalence among the 769 adolescents and young adults reporting ≥ two risky sexual practices when compared with the 1542 adolescents and young adults who reported only one or no risky sexual practices (10% vs. 7%, respectively; *p* = 0.07). Among the 769 adolescents and young adults who reported engaging in ≥ two risky sexual practices, 45% had documentation of HIV testing in the past 12 months and this testing prevalence was significantly higher than the prevalence of 40% among adolescents and young adults reporting one or no risky sexual practices (*p* < 0.0001).

## Discussion

Female adolescents and young adults aged 16–24 years in Botswana were significantly more likely than their male counterparts to report being sexually active, and to report inconsistent condom use, engagement in transactional sex and participation in intergenerational sex with a partner ≥ 10 years older. These practices have been shown to be associated with higher risk of HIV acquisition.^9,10^ With female adolescents and young adults experiencing the highest HIV incidence globally, programmes targeting structural and behavioural drivers of these practices could significantly reduce overall HIV incidence in high burden HIV settings.

Adolescent and young adult females participating in the YaTsie study were more likely to be HIV-infected than their male counterparts. This is consistent with current HIV epidemiological patterns with adolescent and young adult females having the fastest growing incidence of HIV.^11^ In addition, the higher frequency of intergenerational sex reported by young women in the YaTsie study compared to young men has also been described in other high HIV prevalence settings such as Zimbabwe^11^ and Uganda.^12^ Similarly, the association between intergenerational sex and higher HIV prevalence in young women that we found has been observed elsewhere.^12^ Prevention programmes should be specifically tailored to address the unique behaviours or social challenges that place young women at a higher risk for HIV acquisition (transactional sex, intergenerational sex, inconsistent condom use). For example, in our cohort, food insecurity and lack of cell phone were associated with higher prevalence of transactional sex among females. Cash transfer programmes have been shown to decrease transactional sex and intergenerational sex in adolescent females.^13,14^ There is also some evidence pointing to an association between educational level and HIV acquisition.^15,16^ De Neve et al. found that increasing education through to secondary level resulted in a reduction of absolute cumulative risk of HIV infection of 8.1 percentage points (*p* = 0.008).^16^ PEPFAR has adopted educational subsidies as a component of its DREAMS programme to address structural drivers of HIV risk among young females.^17^ While cash transfers, educational subsidies or block grants may not address all risky sexual practices, integration of these types of incentives into existing combination prevention programming may contribute to reductions in HIV acquisition in adolescent females.

In our survey, males were more likely to report sexual debut before 15 years of age, a finding consistent with the Botswana AIDS Impact Survey (BAIS IV) 2013.^18^ Mhalu et al. observed similar differences in self-reported early sexual debut prior to age 15 among Tanzanian adolescents and young adult males and females aged 15–24 years living with HIV, but with a strikingly higher prevalence of early sexual debut at 85% for males compared to 68% for females (*p* = 0.05).^19^ Preventive programming may lead to a decline in age at sexual debut. For example, from 1999 to 2016 in Uganda, the number of participants in the 15–19 year age group who reported never having initiated sex increased from 35% to 56% (*p* < 0.0001) among males and from 28% to 55% among females (*p* < 0.0001),^20^ and decline in early sexual debut among females with concurrent school enrolment achieved significant reduction in HIV infections.^21^ This highlights the value of contextualising prevention programming to gender- and age-specific HIV acquisition risk factors.

The high proportions of both adolescents and young adults reporting secondary education presents an engagement avenue for reinforcing safer sexual practices in young persons. A randomised study in Kenya found that school-based programming resulted in a reduction in unsafe sexual practices among teenagers.^22^ In a pilot programme in Botswana, peer messengers were effective in educating adolescents on the HIV risk associated with intergenerational sex and had an overall impact on sexual behaviours.^23^ In Western Cape, South Africa, a peer-education curriculum designed for adolescents was found to qualitatively improve adolescent self-efficacy in sexual relations and HIV knowledge.^24,25^

Adolescent and young adult females in our cohort were more likely to have participated in HIV testing previously compared with their male counterparts, and pregnant females or those with at least one child were significantly more likely to have participated in HIV testing previously than nulliparous females. This most likely relates to Botswana’s national policy of opt-out HIV testing during antenatal care. There was no significant difference in the proportion of individuals with prior HIV testing experience when comparing adolescent and young adult females without prior pregnancies (62%) and adolescent and young adult males (61%) (*p* = 0.80). Certain strategies may lead to increased HIV testing among adolescents and young adults. For example, the Sustainable East African Research in Community (SEARCH) Trial, a community-based universal test-and-treat trial in Uganda and Kenya offered mobile multi-disease testing, during which participants were screened for hypertension, malaria and HIV.^26^ This multi-faceted mobile approach significantly improved HIV testing uptake among younger participants aged 10–24 years, including very high participation (69%) among young males.

Our study has several limitations. For example, sexual practices were self-reported in a format that required disclosure to a study team member. While our study staff underwent interview training and techniques to promote a non-judgemental, accepting environment through role playing, it is likely that some individuals may not have felt comfortable fully disclosing their sexual practices. Therefore, results may reflect conservative estimates. From its inception, the YaTsie study was structured to evaluate HIV incidence among community members aged 16–24 years. Survey instruments were not administered or HIV testing performed on individuals younger than 16 years of age in the main study and, therefore, our analyses do not include persons < 16 years of age, a population also at risk for HIV acquisition. We asked about the age of sexual debut without providing a specific definition of what constituted sexual debut or inquiring about the consensual nature of the activity. It would be beneficial in future studies to provide respondents with a clear definition and inquire about consensual participation. Our findings may not be generalisable to urban settings, as the YaTsie study communities were located in rural and peri-urban settings. At the YaTsie study inception on an *a priori* basis, we identified sexual practices that would likely place respondents at risk for HIV acquisition or identify individuals at high risk for HIV transmission. This was based on general evidence that has emerged as the HIV epidemic matured. However, we did not include all potential risk factors. Specifically, we did not inquire about whether adolescent and young adult males were having sex with males. Yet, prior surveys have noted that up to 20% of men who have sex with men in Botswana are HIV-infected and nearly 50% of these individuals also reported having female sex partners.^27^ In this analysis, no attempt has been made to correlate the selected sexual practices with actual HIV transmission. As such, we are unable to comment on the quantitative HIV risk associated with the practices included in this analysis. Furthermore, while we use HIV status as a predictor as it relates to intergenerational sex among females and early sexual debut among males, it may actually reflect an outcome, as the timing of HIV acquisition relative to the risky sexual practice was not sought. However, the self-reported sexual practices with differences noted between age groups and within age groups by sex represent a strong starting point to inform HIV prevention programming. Lastly, we acknowledge that there are limitations and biases inherent in a cross-sectional study design.

## Conclusion

In our survey, adolescent and young adult females had a higher prevalence of HIV than males, with a unique set of self-reported risky sexual practices. Structural and behavioural drivers of these risky sexual practices argue for contextualised interventions and prevention programming. Given that female adolescents and young adults are experiencing the highest incidence of new HIV infections globally, prioritising the identification and implementation of efficacious interventions will likely have a significant impact on curtailing the global incidence of HIV. While PEPFAR and the Joint United Nations Programme on HIV/AIDS (UNAIDS) have partnered with host governments to develop and implement programmes focussed on curtailing incident HIV infections among adolescent and young adult females, the specific findings from this study can be used both to inform the further development of these programmes in Botswana and to highlight the importance of contextualising programming to the community and highest risk persons within a community.
